# Supplementary material for: Are carbon emissions trading and green financial instruments synergistic? -Comprehensive quantitative research based on content analysis
Source: PLoS One. 2024 Mar 7;19(3):e0298601. doi: 10.1371/journal.pone.0298601 (PMC10919601; doi:10.1371/journal.pone.0298601)
Supplement: S1 Data — (ZIP) [file pone.0298601.s001.zip › Data/地级市Z_F工作报告.zip!/║■─╧/╓Ω╓▐╩╨/└┤╫╘╣1⁄2╓┌║┼í╛┬φ┐╦╩2╛▌═°í┐_2018.docx]

各位代表：

　　现在，我代表市人民政府向大会报告工作，请予审议，并请各位政协委员提出意见。

　　一、2017年工作回顾

　　过去的一年，是本届政府的开局之年。我们坚持以习近平新时代中国特色社会主义思想为指引，认真贯彻落实新发展理念，在市委的坚强领导下，攻坚克难，真抓实干，全市经济保持平稳健康发展，社会大局和谐稳定。预计全市地区生产总值2700亿元，增长8%，固定资产投资增长13.5%，规模工业增加值增长7.2%，一般公共预算收入增长8%，社会消费品零售总额增长11%，城乡居民收入增长8%。人均GDP跨越1万美元大关。

　　——这一年，我们坚持以迎接、学习、宣传、贯彻党的十九大为工作主线，推动稳增长、促改革、调结构、惠民生、防风险等各项工作取得新进步。培育发展战略性新兴产业、老工业基地转型升级、公立医院改革三项工作获国务院表彰。在2017年中国百强城市排行榜全省6个上榜城市中，排第2位。

　　——这一年，我们加快建设中国动力谷，轨道交通、通用航空、汽车等优势动力产业壮大发展，中国动力谷被誉为中国制造业向中高端升级的典型代表，获得国家层面的充分肯定和认同。

　　——这一年，我们加快建设“一带一部”开放发展先行区，加快“引进来”和“走出去”步伐，成功举办中国企业家年会、轨道交通产业国际高峰论坛、陶瓷产业博览会、服饰产业博览会等重大活动，城市知名度和开放度显著提升。

　　——这一年，我们加快建设老工业城市转型发展示范区，以壮士断腕的决心、背水一战的意志推进清水塘老工业区搬迁改造，147家中小企业关停验收，其中61家企业转型发展，转型升级取得突破性进展。

　　——这一年，我们加快建设城乡统筹发展幸福区，以“敢叫日月换新天”的气概决战脱贫攻坚，有望在全省率先实现整体脱贫，全面建成小康社会。大力实施交通畅通、旧城提质、城市绿荫、碧水蓝天等行动，成功蝉联全国文明城市，以全省第一的成绩通过国家卫生城市复审。

　　一年来，我们主要做了六个方面的工作：

　　（一）着力调结构、兴产业，实体经济加速壮大。现代农业稳步发展。农业增加值增长3.5%，农产品加工总产值增长21%。粮食产量稳定在180万吨以上，新增高档优质稻面积31万亩，建立农业标准化示范基地80个。新增农民合作社127家。建成省级以上畜禽养殖标准化示范场60个，新增农产品“三品一标”认证71个。工业经济提质增效。大力推进中国制造2025试点示范市建设，成功入选国家首批通用航空产业综合示范区、国家首批老工业城市和资源型城市产业转型升级示范区、国家低碳城市试点。新增规模工业企业203家，新开工园区工业项目105个，新引进配套企业106家，实现科技成果转化110项。北汽二工厂、长城电脑、两机重大专项、IGBT产业化二期等一批重点项目，顺利推进或建成投产。加快建设国家自主创新示范区，全球首列智轨列车、12米纯电动智能驾驶客车、石墨烯超级电容器等一批高端新产品诞生。株洲高新区在全国的排名提升至23位，较上年前进5位。株洲经开区挂牌运行。服务业发展提速。第三产业增加值突破千亿大关。成功举办首届神农医药文化产业论坛、湖南夏季乡村旅游节、湖南（株洲）第三届电商峰会等活动。金融业快速发展，飞鹿股份、宏达电子首发上市，上市挂牌企业数量居全省第二。全市接待游客5265万人次，实现旅游总收入463.7亿元，分别增长17%、18%。

　　（二）着力攻项目、扩投资，发展后劲持续增强。项目建设扎实推进。持续开展“项目攻坚年”活动，全市新开工项目4168个，竣工项目3599个，其中重点项目完成投资1200亿元。环线贯通工程、中车物流基地等一批重点项目开工建设，轨道交通城、汽车博览园、湖南移动大数据中心、中国陶瓷谷、东城大道等一批项目加快推进，通用机场、华锐硬质合金、南方宇航高精传动等一批项目竣工投产或建成运营。项目引进力度加大。获批国家智能制造、工业强基等项目13个、制造强省项目38个。通过港洽周、深圳产业对接会、北京项目对接会等招商活动，成功引进恒大健康养生城、华夏幸福、万魔声学等重大项目，合同引资2859亿元。重点招商项目履约率87.3%。要素保障支撑有力。强化精准供地，大力开展征地拆迁“百日攻坚”和集中控违拆违行动，完成集体土地征拆4.3万亩、国有土地房屋征收58.2万平方米，城区拆违116万平方米。强化多元融资，全市新增社会融资760.2亿元，其中新增直接融资471.5亿元，社会融资规模居全省前列。

　　（三）着力破瓶颈、激活力，改革开放纵深推进。供给侧结构性改革持续深化。坚持引促结合、分类推进、关收并举，推进清水塘老工业区搬迁改造，中小企业关停取得决定性胜利，企业转移转型、环境治理、新城建设、产业导入同步推进。钢铁过剩落后产能全部退出，关停退出煤矿、烟花爆竹企业163家。积极化解商品房库存，商品房待售面积下降40%。深入开展“企业帮扶年”活动，推出“24小时见面机制”、第三方机构评估等新举措，全市共收集交办企业各类问题2380个，办结率90%。全面实施正税清费行动，减免企业税费超13亿元。规范政府融资与债务管理，有序推进政府债务偿还和置换。重点改革取得突破。加大“放管服”力度，建设领域投资审批制度改革和“一门式”服务获省首届管理创新奖。在全省率先开展“减证便民”改革，精简幅度超过90%。实现“41证合一”，是全省整合证照最多的市州之一。出台实施“财源建设12条”，财税质量稳步提升。组建金融服务集团，完成农信社改革。土地“三权分置”有序推进，农村土地承包经营权确权登记颁证基本完成。党政领导干部生态环境损害责任追究、绿色信贷管理等改革全面实施。对外开放不断扩大。保税物流中心（B型）验收运营，铁路口岸和国际物流平台加快建设。全年完成进出口总额21.2亿美元，增长28.4%。实际利用外资11.6亿美元，增长10.9%。醴陵市成为全国首个出口电瓷质量安全示范区。“湖南跨贸云”项目落户株洲。中车株机、旗滨集团、联诚集团等海外投资项目有力推进。

　　（四）着力抓建设、强管理，城乡面貌不断改善。中心城区提质升级。湘江七桥、湘江大道一期等建成通车，响石广场改造工程启动建设。航空城、轨道交通城路网加快拉通。“公交都市”“绿色交通城市”创建力度加大，出租汽车行业改革稳步推进。改造小街小巷67条，完成老旧小区专项整治项目10个，改造开工棚户区3.3万户。启动杜甫草堂、白石人家等历史文化特色街区保护工程。完成湘江株洲城区河东段综合治理等70个工程景观建设。建成林荫道路9条，林荫停车场14个。城市管理更加精细。深入推进城管综合执法体制改革，获评全国先进。积极引导社会力量参与城市管理，“西瓜地图”“夜宵地图”等人性化管理获人民网点赞。智慧城管、智能交通、社会治理网格化管理等系统逐步完善。城乡建设步伐加快。“一区一县九镇”多规合一基本完成，武广新城、枫溪新城等片区加快开发。皇图岭镇、白兔潭镇分别获批全国特色小镇和省级美丽乡镇。《株洲市农村村庄规划建设管理条例》顺利实施。硬化农村公路235公里，窄路加宽226公里，改造危桥44座，建设农村招呼站150个。新增城镇管输天然气用户5.1万户、自来水用户4.2万户。兴建各类水利工程1.8万处，解决16.5万农村人口饮水安全问题。环境治理取得实效。全面推行河长制，市县乡村四级河长体系初步建立。扎实推进湘江保护治理“一号重点工程”，259个项目顺利实施。完成3处城市黑臭水体整治和6个工业园区污水处理设施建设，洗水工业园、枫溪污水处理厂建成运行。淘汰黄标车7816台。关停不达标规模养殖场372个。完成生态环保红线划定和267个行政村环境整治。造林18万亩，湘江流域退耕还林还湿试点项目有序推进。中央环保督察交办问题基本整改到位。

　　（五）着力办实事、惠民生，群众福祉日益提升。脱贫攻坚成效显著。大力实施“七个一批”“六大工程”，深入推进专项扶贫、行业扶贫和社会扶贫。5.67万贫困人口实现脱贫，126个贫困村退出，炎陵、茶陵已顺利通过省检，全市贫困发生率由2016年的3.64%下降到1.29%，脱贫攻坚取得决定性成效。“民生100”工程逐一落实。民生支出占财政总支出比重保持在70%以上。新增城镇就业和农村劳动力转移就业任务超额完成。城乡居民大病保险全面实现市级统筹、分级管理，在全省率先实施异地就医联网结算。最低生活保障标准和国家扶贫标准实现“两线合一”。农村五保对象基本医疗费全额减免。切实保障干部工资待遇，企业和机关事业单位退休人员养老待遇同步提升。获批全国居家和社区养老服务改革试点城市。完成农村危房改造9500户。市特殊教育学校建成使用，市属三所职业院校主体工程基本完工。爱心大院、云龙医院、田心医院提质改造有序推进，计划生育工作连续三年全省优秀。创建国家公共文化服务体系示范区通过中期评估，“一宫三馆四中心”全面建设，成功举办炎帝陵省祭、全国老年合唱节、全球华人羽毛球赛等重大文体活动。社会大局和谐稳定。圆满完成村（居）两委换届工作。平安株洲、法治株洲建设深入推进。成功侦破“3·15”维卡币特大网络传销案。第四次捧回“长安杯”。获全国综治工作优秀地市“六连冠”，全省唯一。大力开展安全生产大排查大管控大整治百日行动，妥善处置攸县“5·7”重大中毒窒息事故。食品药品安全监管持续加强。全力应对历史罕见洪涝灾害，取得了未垮一库一堤、无一人因灾伤亡的全面胜利。出色完成党的十九大等重大活动的信访维稳安保工作。积极开展军民共建，全面完成民兵训练、兵员征集任务，国防动员能力不断加强。

　　（六）着力转职能、树形象，政府自身建设全面加强。突出制度建设，进一步规范政府权力配置和运行。“马上就办、真抓实干”活动扎实开展。以“十个100”为抓手，严格实施重点工作月度考核，驰而不息推动工作落实。全面贯彻执行法治政府建设实施纲要。深入推进“两学一做”学习教育常态化制度化。严格落实全面从严治党“两个责任”，廉政建设和反腐败斗争不断加强。认真办理市长热线和市长信箱，积极回应群众诉求。坚持重大问题及时向市委汇报，全面执行市人大及其常委会法规、决议和决定，自觉在市人大、市政协和社会各界监督下开展工作，充分吸纳各民主党派、工商联、无党派人士和社会各界的意见、建议。积极落实市人大常委会关于中国动力谷建设等6个审议意见。认真办理人大代表建议297件、政协提案344件，解决率不断提升。

　　与此同时，统计、审计、工商、质监、检验检疫、外事侨务、人防、国安、移民、供销、广电、新闻出版、档案、地方志、气象、水文、民族宗教、住房公积金、对口支援、妇女儿童、残联、文联、红十字、贸促、侨联、对台等工作都有新进步。

　　各位代表！过去的一年，我们付出了艰辛，流下了汗水，干了一些难事，办了一些大事。这些成绩，是在经济形势复杂多变、经济增长动力不足的背景下取得的，是在清水塘搬迁改造、煤炭烟花爆竹去产能力度空前、老工业城市转型阵痛加剧的条件下取得的，是在抗洪抢险、环境治理、脱贫攻坚等多重压力叠加的情况下取得的。这是市委正确领导的结果，是市人大、市政协监督与支持的结果，饱含了各位代表、各位委员的智慧和奉献，凝聚了全市人民的心血和力量。在此，我代表市人民政府，向全市广大干部群众，向人大代表、政协委员，向各民主党派、工商联、无党派人士和人民团体，向驻株部队官兵以及中央、省驻株单位，向离退休老同志，向所有关心支持株洲发展的各界朋友，表示崇高的敬意和衷心的感谢!

　　在总结成绩的同时，我们也清醒看到存在的矛盾和问题：一是经济下行和结构调整的压力依然较大，产业发展新不足以补旧、小不足以补大、增不足以补减，新动能培育有待进一步加强。二是项目储备不多，特别是产业项目偏少，发展后劲不足。发展的不平衡问题较为突出，县域经济支撑还不够有力。三是经济社会发展中的风险不容忽视，防范化解债务风险的压力较大，安全生产、征地拆迁、信访维稳、非法集资等领域风险防控还需加强。四是部分干部思想观念、专业素养、创新意识、担当精神与新时代的发展要求还不适应，少数干部存在为官不为、精神懈怠的问题。对此，我们将采取有力措施，加以解决。

　　二、2018年主要目标和任务

　　2018年是全面贯彻党的十九大精神的开局之年。党的十九大立足社会主要矛盾新变化、经济发展新常态、国际国内新形势，确立了新思想，明确了新目标，提出了新要求，为我们指明了前进方向，提供了根本遵循。2018年是改革开放40周年。从改革发端，到深化改革，再到全面深化改革，改革开放为发展积聚了更大动力、释放了更强活力，我们将逢山开路，遇水架桥，将改革开放进行到底。2018年是我市基本现代化的起步之年。我们正处在实现整体脱贫、全面建成小康社会、开启基本现代化新征程的崭新发展阶段，将朝着经济现代化、政治现代化、文化现代化、社会现代化、生态文明现代化的道路迈出坚定有力的新步伐。“新故相推，日生不滞。”新时代的伟大征程已经开启，我们要勇做新时代的坚定者、奋进者、搏击者，不驰于空想，不鹜于虚声，一步一个脚印，在新时代的宏阔画卷上绘就更加辉煌的图景，大踏步迈向更加美好的未来！

　　2018年政府工作的指导思想是：全面贯彻党的十九大精神，以习近平新时代中国特色社会主义思想为指导，认真落实省委、省政府和市委的决策部署，坚持稳中求进、进中争先工作总基调，坚持新发展理念，紧扣社会主要矛盾变化，按照高质量发展要求和更好发挥“四个作用”要求，坚持以供给侧结构性改革为主线，切实落实省委创新引领、开放崛起战略，以产业项目建设年为抓手，着力构建现代化经济体系，统筹做好稳增长、促改革、调结构、惠民生、防风险各项工作，实现整体脱贫和全面建成小康社会目标，开启基本现代化新征程。

　　今年发展的主要预期目标是：地区生产总值增长8.5%左右；规模工业增加值增长7.5%；全社会固定资产投资增长12%；社会消费品零售总额增长11%；一般公共预算收入增长7%左右；城乡居民收入高于经济增长；完成省下达的节能减排任务。

　　各位代表！新时代最鲜明的特征是，经济由高速增长阶段向高质量发展阶段转变。做好今年政府工作，要致力于推动高质量发展，重点把握以下几点：

　　一是坚持“稳中求进、进中争先”总基调，准确把握工作节奏和力度。审时度势，尊重规律，该稳的稳住，该进的进好，该争的力争。“稳”是主基调，主要是稳预期、稳增长、防风险、守底线。“进”是主方向，重点在产业升级、改革创新、开放发展、乡村振兴等关键领域积极进取。“争先”是主要求，是株洲发挥“四个作用”、实现“两个走在前列”的应有之义、应担之责。按照这一总基调，我们确立了保持8.5%左右的增长速度，既体现了省委、省政府的要求，也体现了自我加压、奋勇争先的坚定决心。

　　二是推动“三大变革”，着力提升经济发展的质量和效益。高质量发展，就是能够很好满足人民日益增长的美好生活需要的发展，是体现新发展理念的发展。要坚持质量第一、效益优先，以提高供给体系质量为主攻方向，深化供给侧结构性改革。坚持以抓改革为重点推动动力变革、以优服务为重点推动效率变革、以促创新为重点推动质量变革，加快形成推动高质量发展的指标体系、政策体系、绩效评价和政绩考核体系，加快实现向高质量发展的根本转变。

　　三是开展“产业项目建设年”，突出抓重点、补短板、强弱项。抓经济的关键是抓产业、抓项目。把“产业项目建设年”作为今年经济工作的主抓手，既落实上级要求，又符合株洲实际，是我市近几年推进“项目攻坚年”和“企业帮扶年”的提升和深化。我们将全面对接全省“产业项目建设年”提出的“5个100”工程，重点推进“五个一批”计划，聚力实施“五大行动”，加快构建实体经济、科技创新、现代金融、人力资源协同发展的产业体系。

　　新时代开启新征程，新使命呼唤新作为。我们将以永不懈怠的精神、一往无前的勇气，凝聚全市上下同心共筑中国梦的磅礴力量，下定决心，不怕牺牲，排除万难，去争取建设富饶、美丽、幸福新株洲的新胜利！

　　三、2018年重点工作

　　围绕以上目标和要求，主要做好以下七个方面工作：

　　（一）加快产业转型升级

　　实施规划引领行动。持续推进工业“1093”行动，制定中国动力谷实施方案，编制新材料、先进陶瓷、生物医药等新兴产业规划，完善产业规划体系，优化产业布局。围绕11个优势产业链，针对关键节点和薄弱环节，明确任务书、路线图、时间表，着力补链、强链、延链。优先策划主机引进项目，年内引进1家以上。注重产业规划融合，加快建设大数据、工业云平台，开展三年千家企业“上云”行动，积极培育智能制造、网络众包、云制造等新技术、新模式、新业态。加快南方军民融合协同创新中心建设，促进军民融合。

　　实施项目攻坚行动。总结推广“项目攻坚年”成功经验，重点攻坚项目前期、招商引资、要素保障等关键环节，提高投资效益，力争全年产业投资过1000 亿元，占固定资产投资40%以上。对接落实省“5个100”建设任务，充分发挥产业优势，积极争取国省项目和资金支持，着力策划引进一批项目，强化产业发展支撑。重点实施一批重大产业项目：启动商用车车桥及高速齿轮制造、株冶锌基材料等项目建设；推进孚能新能源动力电池、高速道岔产业化及智能制造等项目建设；确保格斯特航空发动机零部件生产及配套一期、中车时代电动汽车系统产业化等项目竣工投产。创新融资手段，设立产业发展引导基金，支持政策性融资担保公司做大做强，全年新增社会融资400亿元以上。加大征拆力度，完成集体土地征拆3.18万亩、国有土地房屋征收40万平方米。

　　实施园区升级行动。大力开展园区攻坚三年大会战。力争园区引进项目当年履约率70%以上，当年开工率50%以上，园区主特产业占园区经济总量达到60%以上。健全园区管理体制，赋予园区在项目审批、土地开发、财政收支等方面更多的自主权。进一步优化园区产业布局，突出差异化、特色化发展，引导和支持优势产业链向县市工业园区延伸，加快建设数据湖、信息港、华夏幸福云龙产业新城、醴陵电力电瓷电器产业园、攸县长明新能源基地等一批产业发展平台。进一步强化园区建设管理，完善生产和生活配套，重点整治田心片区、董家塅片区等一批老工业区周边环境。加大园区土地储备和开发力度，切实提高标准厂房的使用率。创造条件支持醴陵经开区、株洲经开区申报国家级经开区。推动株洲经开区与长沙的产城对接，发挥株洲在长株潭一体化中的主动作用。

　　实施设施配套行动。坚持以“支持产业发展、满足企业需要、方便员工生活”为导向，完善水、电、路、气、讯等基础设施，加强教育、医疗、文化等公共服务设施建设，提升产业配套服务功能，促进产城融合。大力发展现代金融、现代物流、职业教育和人力资源等生产性服务业，加快株洲电商城、中车物流等项目建设，推进金融集聚区发展，打造一批电子商务平台、物流配送平台和金融服务平台，推动生产性服务业与制造业相互促进、融合发展。

　　实施环境优化行动。着眼于提升城市软实力和核心竞争力，把优化发展环境摆在更加突出的位置，为实现高质量发展提供更加有力的支撑。优化政策环境，坚决落实产业扶持政策，完善相关产业配套政策，加强诚信政府建设，兑现已出台政策。优化服务环境，加大企业帮扶力度，进一步优化审批程序，完善重大项目联系服务机制，围绕企业需求提供快速、精准和一站式服务。优化法治环境，规范涉企检查行为，加大对强买强卖、阻工揽工等违法行为的打击力度，切实维护企业的合法权益。构建“亲”“清”新型政商关系，既要积极引进新企业，又要支持本土企业发展壮大，努力营造亲商安商富商的浓厚氛围。

　　（二）坚决打好“三大攻坚战”

　　打好防范化解重大风险攻坚战。坚持问题导向、底线思维，加强重点行业、重点领域的风险防范化解。摸清政府债务底数，进一步规范政府举债行为，严格依法依规实施PPP、政府购买服务，科学制定偿债计划，严格控制增量，逐步消化存量。积极推进混合所有制改革和政府投融资公司市场化转型。严厉打击非法集资、非法揽存等违法违规金融活动，严密防控民间融资、各类交易场所、互联网金融等金融风险，坚决守住不发生系统性风险的底线。健全社会矛盾排查预警、重大决策社会稳定风险评估、矛盾多元化解机制，加快建设“综治中心+网格化+雪亮工程”的一体化综合平台。

　　打好精准脱贫攻坚战。瞄准特定贫困群众精准帮扶，聚焦深度贫困地区精准发力，确保现行标准下的脱贫质量，确保贫困县脱贫摘帽一举成功，确保省下达的减贫人口任务圆满完成。完善脱贫攻坚长效机制，全面落实“四个不摘和四个只增不减”的要求，持续做好产业、就业、教育、健康、住房安全、兜底扶贫等工作，让有劳动能力的贫困户家家有就业，让特殊群体贫困户人人有保障，确保稳定脱贫不返贫。

　　打好污染防治攻坚战。深入开展湘江保护治理第二个“三年行动计划”，压实四级河（湖）长管水治水护水职责，将环保网格化监管延伸到水面。完成枫溪港、陈埠港、凿石港黑臭水体整治，加快重点建制镇污水处理厂建设。全面完成水生态文明城市建设试点任务。切实加强企业达标排放、扬尘污染管控、餐饮油烟整治等工作，不断提高空气质量，打赢蓝天保卫战。抓好世行、亚投行土壤治理项目、铜霞路以南地块生态修复，完成国家环境污染第三方治理试点任务。推进生态绿心地区工业企业退出，完善生态补偿机制。深化农村人居环境整治，实施农业面源污染综合防治工程，加快污染耕地修复治理。

　　（三）深化改革创新

　　深化供给侧结构性改革。重点在“破、立、降”上下功夫，着力提升供给体系质量。大力破除无效供给，全面处置“僵尸企业”，淘汰煤炭、烟花鞭炮等行业落后过剩产能。坚定不移、全面关停株冶及配套企业，确保清水塘老工业区搬迁改造取得标志性进展。大力培育新动能，积极导入新业态，启动建设清水塘科技创新园。坚持“房子是用来住的、不是用来炒的”，加快建立多主体供给、多渠道保障、租购并举的住房制度，加大非住宅商品房去库存力度，促进房地产市场健康平稳发展。调整和公布涉企收费目录清单，全面清理规范中介服务，进一步降低企业制度性交易成本和融资、人力、物流等成本。毫不动摇鼓励、支持、引导非公有制经济发展，制定民间投资引导目录，放宽民间投资领域的范围。弘扬优秀企业家精神，完善公平竞争环境，促进民营企业加快发展。

　　推进重点领域改革。深化“放管服”改革，把经济管理权放到市场最需要的地方，把社会管理权放到群众最方便的地方。结合“智慧株洲”建设，积极推广“一门式”政务服务，重点推进“最多跑一次”改革，让数据“多跑路”、群众“少跑腿”。深化财税体制改革，完善预算管理制度，全面实施绩效管理，提高财政收入质量和财政资金效益，做好增值税、消费税、个人所得税等配套改革。深化金融体制改革，增强金融服务实体经济能力，实施上市公司培育工程，利用好多层次资本市场。深化农村综合改革，完善“三权分置”制度、农民闲置宅基地和闲置农房政策，推进农村集体产权制度改革试点。深化生态文明制度改革，全面开展第三次土地资源大调查，启动自然资源确权登记。深入推进统计管理体制改革，抓好第四次全国经济普查工作。统筹推进监察、环保、教育、医疗、文化、市场监管、供销合作、安全生产监管等领域改革，力争在两型社会、新型城镇化等“国字号”改革试点上形成新经验新模式。

　　激发创新创业活力。统筹推进国家自主创新示范区、国家创新驱动助力工程示范城市以及国家创新型城市建设。着力建设一批“双创”平台，推动IGBT技术创新中心、轨道交通制造业国家级创新中心建设，支持高新区申报国家级双创示范基地，新增孵化器面积20万平方米以上。着力推进一批重大科技创新项目，重点实施两机重大专项、中车电器技术与材料工程研究院等项目。加快组建10亿元以上的科技成果转化基金，全年推进100项以上科技成果转化。着力推进一批重大产品创新，重点实施跨坐式单轨车辆、小型通用飞机涡桨发动机、碳陶新材料等研发生产项目。着力培育引进一批科技创新人才，落实国家“千人计划”、省“芙蓉人才计划”和市“人才新政30条”，实施高层次人才集聚工程，设立全球动力产业专家人才库，建设100名科技领军人才队伍。支持企业建设院士工作站、博士后工作站等人才集聚平台，新建一批高端创新团队。弘扬工匠精神，加大高素质技能人才培养力度，促进人才队伍与产业发展深度融合。加强名品名牌建设，确保全国质量强市示范城市创建通过验收。策划引进国家级检测认证机构，参与行业标准体系制定，提升株洲制造话语权。

　　（四）加大开放发展力度

　　加强招商引资。大力推进“对接500强、提升产业链”行动和“对接湘商会、建设新家乡”行动，完善招商项目库和3个500强信息库，掀起大招商、招大商热潮。依托“沪洽周”“厦洽会”等平台，加强以商招商、产业链招商、科技招商和资本招商。充分利用株洲异地商会等各类湘商资源，引进一批湘商回株发展。大力发展会展经济，探索运用市场化模式，办好五大产业会展和第十五届国际茶文化研讨会。建立重大招商引资项目服务联动协调机制和项目代办机制，以钉钉子的精神招商引资，对看准的大项目、好项目，快速决策，盯住不放，确保落地。力争全年实际利用外资增长14%以上，利用内资增长18%以上，签约亿元以上重大项目100个以上。

　　壮大外经外贸。大力推进“对接新丝路、推动走出去”行动，全面实施外经外贸“破零倍增”计划，力争全年进出口总额增长15%以上。加大“引进来”力度，加强与阿里国际站和中国国际电子商务的合作，引进高端电子商务物流商，建设跨境电商订单履约仓和备货集货中转中心。加快“走出去”步伐，打造株洲硬质合金、醴陵陶瓷、芦淞服饰等区域性国际品牌，加强通关、结汇、退税、物流、信保等服务，支持优势产业、优秀企业和优质产品走出去。

　　夯实开放平台。大力推进“对接自贸区、提升大平台”行动和“对接北上广、优化大环境”行动。建立健全口岸经济区管理机构，完善株洲保税物流中心功能，启动铁路口岸、外贸综合服务平台、口岸经济区建设。争取“湘欧”快线延伸至我市，加快打通水、陆、空开放通道。支持一批外贸平台企业开展一站式综合服务，引进一批供应链综合服务商。推动贸易便利化改革，落实关检“三个一”深度合作，全面实现国际贸易“单一窗口”，推进全国通关一体化合作。

　　（五）提升新型城镇化发展水平

　　完善城市综合功能。开展“城市双修”，不断提升城市承载能力。大力实施交通畅通行动，启动湘江八桥、铁东路北段全线路基工程建设，加快火车站改建等项目进度，拉通北环线C段、中车大道等一批骨干道路和园区道路，打通航空大道、黄山西路、劳动支路等一批城市“断头路”，疏通响石广场等一批交通堵点。启动建设路、新华路 BRT 项目建设。大力实施城市绿荫行动，加快推进神农生态带、雪峰岭公园、云峰森林植物园等项目建设。大力实施旧城提质行动，加快“两岸一线”景观靓化、历史文化街区、老旧小区等项目建设。实施绿化美化亮化净化工程，进一步提升城市品质品位。启动“十大公园十大湖”“一江八港六走廊”建设，加快推进地下综合管廊和海绵城市建设。

　　推进城市管理升级。深化城市管理和综合执法体制改革，全面实施《株洲市城市综合管理条例》，强化环卫保洁、园林绿化、市政养护、渣土运输等精细管理，巩固“创文”“创卫”成果。加快餐厨废弃物资源化利用、垃圾焚烧发电厂二期、南郊垃圾填埋场封场等项目建设。持续抓好查违拆违控违，完成城区拆违100万平方米。深入开展“文明城管”行动，推进智慧城管升级，积极探索“城管+律师”执法新模式，提升城市管理人性化、智能化、法治化水平。

　　加快特色城镇建设。深化国家新型城镇化综合试点，大力推进城乡一体化发展。加快攸县撤县设市、株洲县撤县设区步伐。统筹城市和城镇规划布局，推广首批12个乡镇村庄规划建设管理行政审批试点经验。突出工业、农业、旅游、文化、康养、金融等业态融合，重点培育20个特色城镇，形成各具特色的小城镇建设发展模式。加快扩权强镇步伐，将规划、建设、城管等行政执法权授予特色镇行使。通过“以奖代补”竞争机制配套城镇基础设施资金。运用PPP、财政贴息、发行项目收益债等多种方式，激励社会资本投入，释放城镇建设潜力。

　　（六）大力实施乡村振兴战略

　　加快现代农业发展。深化农业供给侧结构性改革，坚持质量兴农、绿色兴农，加快推进农业由增产导向转向提质导向，构建现代农业产业体系、生产体系、经营体系。突出结构调整，大力发展高档优质稻，推进经济作物提质增效，完成省里下达的农业结构调整指导性任务。重点推进中华茶祖文化产业园、万樟园林、炎陵白鹅等产业项目建设。加快农产品加工业转型升级，新增市级以上龙头企业20家，农产品加工业总产值增长15%以上。突出品牌提升，推进“三品一标”产品认证工作，支持“炎陵黄桃”“皇图岭辣椒”等申请国家地理标志农产品。加大农业标准化示范基地创建和质量溯源体系建设力度，打造一批农业标准化基地和农产品质量安全追溯点。突出新型主体培育，发展适度规模经营，培育一批农民合作社示范社和示范家庭农场。依托农业科技研发平台，建设一批现代农业科技示范园和特色产业园。坚持全景式打造、全产业发展、全方位服务、全社会参与，大力发展全域旅游，加快酒埠江、九郎山、云阳山、神农谷、大京等旅游景区建设，创建炎陵、醴陵全域旅游示范县和炎帝陵国家5A级景区，打造大湘东旅游带。

　　加强农村基础设施建设。坚持以工补农、以城带乡，把公共基础设施建设的重点放在农村，推动农村基础设施建设提档升级。推进茶界高速、渌水航道、S205、S207等项目前期工作。加快S105、东城大道、长株攸快速通道等项目建设，力争莲株高速全线建成通车，确保航电枢纽二线船闸竣工通航。推进“四好农村路”建设，完成自然村通水泥（沥青）路590公里，提质改造农村公路288公里，完成安保工程400公里、危桥改造30座。连片推进农田水利基础设施建设，完成酒埠江大型水库、龙潭中型水库年度除险加固任务和102座小Ⅱ型水库除险加固。推进健康乡村建设，持续改善农村人居环境，大力实施“一廊十镇百村”美丽乡村示范工程，推进村容村貌综合整治，开展村庄规划建设管理试点，整治农村空心破败房。加强网络基础设施建设，实现“三网”融合全覆盖。推进“气化株洲”长输管线建设，开展“气化村镇”试点建设。

　　创新乡村治理体系。推动社会治理重心向基层下移，创新基层民主管理模式，健全自治、法治、德治相结合的乡村治理体系。提高基层组织凝聚力和战斗力，选好管好用好农村基层组织带头人，培育一批专业人才，扶持一批乡村工匠，培养造就一支懂农业、爱农村、爱农民的“三农”工作队伍。健全覆盖城乡居民的公共法律服务体系，加大法律援助力度，切实提高基层法治水平。严肃查处侵犯农民利益的“微腐败”，建设平安乡村。加强农村思想道德建设，传承发展提升农村优秀传统文化，加强农村公共文化建设，开展移风易俗行动，不断提高乡村社会文明程度。

　　（七）持续保障和改善民生

　　深入实施“民生100”工程。坚持把满足人民日益增长的美好生活需要，作为政府工作的责任、动力和取向。在全面完成各项民生指标任务的同时，着力解决一批老百姓的操心事、烦心事：深入推进“厕所革命”，全年新建改建120座公共厕所；改造城市棚户区2.24万户，改造农村危旧土坯房6156户，着力解决困难群众住房难题；开展居民小区违章建筑、消防安全、房屋质量、电梯质量安全等综合治理，加强小区物业规范化管理；修通和改造47条支路及小街小巷；加快“互联网+停车”功能停车信息系统建设，建成一批公共停车场，缓解城市停车难；加大重点拥堵路段改造力度，优化交通秩序管理，打通一批交通堵点；解决11万农村人口饮水不安全问题；继续推进一批农贸市场的提质改造；实施60所农村学校“改薄”工程，改善义务教育办学条件。

　　促进社会事业发展。加快教育强市建设，着力解决学前教育资源不足、中小学生课外负担重、“超大班额”等突出问题。完成南方中学新校主体工程建设，提质改造市一中、三中、八中，推进航空产业职院建设，加快建设铁道职院，确保工业学校、幼师学校和汽车工程职院秋季搬迁开学。支持湖南工业大学内涵建设、特色发展，加快湖南工业大学醴陵陶瓷学院建设。推动民办学校分类登记和管理，扩大普职融通范围。加强教师队伍建设，实施乡村教师支持计划和教师成长幸福工程。深入推进健康株洲建设，深化公立医院配套改革，全面建立分级诊疗，实施第二批临床医学重点专科建设项目，推进平安医院建设，加快基层公共服务能力提升，促进公共卫生服务均等化。争取湖湘中医药博物馆落户株洲。完成国家公共文化服务体系示范区创建。加快市博物馆、美术馆搬迁，完成市群艺馆改扩建，启动市图书馆新馆和文化市场数字化监管平台建设。推动湖南工业博物馆和工业遗址公园建设。全面铺开国家文化消费试点工作。积极参加省第六届艺术节、省第十三届运动会。

　　加大社会保障力度。深入实施就业优先战略和更加积极的就业创业政策，确保全年新增城镇就业5万人、农村劳动力转移就业1.25万人。稳步实施全民参保计划，确保全年新增企业养老保险参保3万人。健全低保标准增长机制，完善社会救助体系，最低生活保障月人均补差提高10%以上。对80岁以上老人，实行高龄补贴扩面提标。新增公积金建制人数1.5万。推动医疗救助与基本医疗保险、大病保险有效衔接，完成9.05万农村适龄妇女“两癌”免费检查。积极应对人口老龄化，推进医养结合，鼓励社会资本进入养老领域，努力打造居家和社区养老服务圈。

　　加强和创新社会治理。构建社会治安立体化防控体系，加快“智慧株洲”电子眼联网项目建设，降压发案数，提高破案率，重点打击黄赌毒、黑拐骗，提升人民群众安全感。深入开展安全生产隐患排查治理，确保安全生产形势平稳有序。开展信访维稳“三无”创建活动。完善突发事件应急管理机制，增强应急处置能力。深入开展国家食品安全示范城市创建，确保人民群众“舌尖上的安全”。做好民族宗教工作。加强国防动员和民兵预备役工作，统筹推进人防及军事设施保护、管理和建设，深入开展军民共建。

　　各位代表！“幸福都是奋斗出来的。”走进新时代，开启新征程，实现新目标，不是喊喊口号，做做样子就能完成的。我们必须俯下身子，撸起袖子，切实加强自身建设，着力打造“法治、诚信、创新、效能、廉洁”政府，为实现全市人民的共同愿景付出更为艰辛的努力。必须忠诚坚定。忠诚于党，增强“四个意识”，提高政治站位，时刻不忘出发志、不失主心骨、不丢方向盘，坚定不移执行党的路线、方针、政策，坚定不移落实中央和省委、市委的决策部署。忠诚于法，深入推进法治政府建设，运用法治思维、法治方式、法治手段行使权力、履行职责，依法接受人大法律监督和工作监督，自觉接受政协民主监督和社会公众监督。忠诚为民，坚持民生优先，不忘公仆之心，永葆民本情怀，利民之事，丝发必兴；厉民之事，毫末必去。必须创新进取。以敢闯敢试的勇气，自我革新的状态，勇于变革、勇于创新，永不僵化、永不停滞。按照党的十九大提出的增强“八种本领”的要求，大兴学习之风、调研之风，不断培育专业精神，提高适应新时代、实现新目标、落实新部署的专业水平。必须务实担当。大力弘扬“火车头”精神，深入开展“马上就办、真抓实干”活动，以真抓的实劲、敢抓的狠劲、善抓的巧劲、常抓的韧劲抓落实，干事业。勇于挑最重的担子，敢于啃最硬的骨头，善于接最烫手的山芋，善谋善断、善作善成。必须清正廉洁。严格落实全面从严治党责任，驰而不息改作风、纠“四风”，重点整治表态多调门高、行动少落实差等问题。加强对重点领域、关键环节的监管，实现公共资金、国有资产、国有资源和领导干部经济责任审计全覆盖，做到干部清正、政府清廉、政治清明。

　　各位代表！人民对美好生活的向往，就是我们的奋斗目标。让我们更加紧密地团结在以习近平同志为核心的党中央周围，坚持以党的十九大精神为指引，在省委、省政府和市委的坚强领导下，不忘初心，牢记使命，奋勇前行，为加快建成“一谷三区”、实现“两个走在前列”而继续不懈努力！
